# 401-Gene Signature of Myocardial Dysfunction in Human Heart Failure: A Transcriptomic Analysis

**DOI:** 10.7759/cureus.91745

**Published:** 2025-09-06

**Authors:** Kiki J Estes-Schmalzl, Amy J Marcano-Reik, Kristin M Lefebvre

**Affiliations:** 1 Clinical Research, University of Jamestown, Fargo, USA

**Keywords:** biomarkers, cardiac remodeling, differential gene expression, heart failure, transcriptomics

## Abstract

Background

Heart failure is a complex clinical syndrome characterized by the molecular remodeling of myocardial tissue that significantly impacts global health outcomes. Transcriptomic analysis offers powerful tools to identify disease-specific gene expression signatures and potential therapeutic targets.

Methods

We analyzed publicly available gene expression data from the Gene Expression Omnibus (GEO) dataset GSE57345, comprising 313 left ventricular tissue samples (295 controls, 18 heart failure) profiled using Affymetrix Human Gene 1.1 ST Arrays (Thermo Fisher Scientific, Waltham, Massachusetts, United States). Differential expression analysis was performed using limma with Benjamini-Hochberg multiple testing correction.

Results

We identified 401 significantly differentially expressed genes (adjusted p<0.05) between heart failure and control samples. The molecular signature showed balanced dysregulation with 198 genes upregulated and 203 genes downregulated in heart failure. Pathway analysis revealed significant enrichment in mitochondrial dysfunction, immune activation, and extracellular matrix remodeling pathways. Top dysregulated genes included AIDC1, SECA1, PVRL2, PLD1, and KTR3CL3, with high statistical significance (p<1×10⁻⁶) despite modest fold changes characteristic of complex disease pathophysiology. Cross-validation with established heart failure signatures showed significant overlap (31.7% concordance), confirming biological relevance.

Conclusions

This 401-gene transcriptomic signature provides insights into heart failure molecular mechanisms and represents a potential resource for biomarker development and therapeutic target identification. The balanced pattern of gene dysregulation reflects the comprehensive transcriptional remodeling underlying cardiac dysfunction and supports precision medicine approaches for heart failure diagnosis and treatment stratification.

## Introduction

Heart failure is a complex clinical syndrome characterized by the heart's inability to pump sufficient blood to meet metabolic demands, representing a significant healthcare burden with substantial hospitalization costs and clinical impact [1]. The molecular pathophysiology involves extensive myocardial remodeling, including alterations in contractile proteins, energy metabolism, inflammatory pathways, and extracellular matrix composition.

Recent advances in transcriptomic profiling, the study of all the RNA molecules expressed in a cell or tissue, have revolutionized our understanding of heart failure mechanisms. A comprehensive RNA-sequencing study of 64 human left ventricular samples revealed that while dilated cardiomyopathy (DCM) and ischemic cardiomyopathy (ICM) share common heart failure pathways, they exhibit distinct etiology-specific signatures, with dysfunctional cell-cell and cell-matrix adhesion characterizing DCM and activated immune pathways typifying ICM [2]. A meta-analysis of 16 publicly available studies comprising 653 heart failure and 263 healthy left-ventricle biopsies established a consensus transcriptional signature independent of technical biases [3]. This consensus approach represents a critical advancement in heart failure research, as it demonstrates the reproducibility of molecular signatures across diverse patient populations and technical platforms, providing a robust foundation for biomarker development and therapeutic target identification. The establishment of reproducible transcriptomic signatures holds significant promise for precision medicine applications, potentially enabling clinicians to stratify patients based on molecular phenotypes, predict treatment responses, and identify novel therapeutic pathways that could transform heart failure management from a one-size-fits-all approach to personalized, molecularly guided interventions.

Heart failure with preserved ejection fraction (HFpEF) represents a growing clinical and public health challenge, accounting for over 50% of all heart failure cases worldwide. The clinical burden of HFpEF is substantial and growing, with prevalence increasing dramatically with age, particularly among women. Despite advances in cardiovascular care, HFpEF carries a mortality rate comparable to many cancers, with five-year survival rates of approximately 35-50%. This systemic inflammatory condition, driven by comorbidities, results in coronary microvascular dysfunction and myocardial remodeling distinct from HFrEF. Despite its prevalence and poor prognosis, HFpEF remains underdiagnosed and undertreated compared to heart failure with reduced ejection fraction (HFrEF), primarily due to its diagnostic complexity, its clinical heterogeneity, and the absence of a universally accepted gold standard [4-8]. Accurate diagnosis of HFpEF is critical, as delayed or missed identification can exacerbate morbidity, hospital readmissions, and healthcare disparities, particularly among socially vulnerable populations [7,8].

Mitochondrial dysfunction as a central mechanism

Emerging evidence demonstrates that mitochondrial dysfunction represents a fundamental mechanism in heart failure pathophysiology [9]. This dysfunction leads to progressive decline in bioenergetic reserve capacity, involving a shift from mitochondrial fatty acid oxidation to glycolytic pathways and contributing to disease progression [10]. Recent transcriptomic analyses of oxidative phosphorylation (OXPHOS) complexes in human cardiac tissue revealed 28 altered genes in heart failure patients, with greater deregulation in ICM and significant overexpression of complex V elements [11]. Mechanistic studies have revealed that cardiovascular aging involves defects predominantly confined to interfibrillar mitochondria, with complex III and IV dysfunction manifesting as decreased oxidative phosphorylation, reduced ATP production, and increased reactive oxygen species generation [12].

Disease-specific transcriptomic signatures

The concept of a "final common pathway" in heart failure has been challenged by findings showing etiology-specific molecular signatures [2]. This paradigm shift has profound implications for understanding heart failure heterogeneity, particularly differences between HFrEF and HFpEF. Analysis of HFpEF revealed distinctive transcriptomic signatures with 1882 upregulated and 2593 downregulated genes compared to controls, including unique mitochondrial ATP synthesis/electron transport pathways [13]. Single-cell transcriptomics further revealed that while cardiomyocytes converge toward common disease-associated states, fibroblasts and myeloid cells undergo dramatic diversification in heart failure [14]. These findings underscore the importance of understanding cell type-specific responses and disease-specific mechanisms, potentially explaining why therapeutic strategies effective in HFrEF have largely failed in HFpEF trials and highlighting the need for precision medicine approaches tailored to specific heart failure phenotypes.

This study aimed to identify differentially expressed genes in human heart failure using robust bioinformatics methods, providing insights into molecular mechanisms and contributing to evidence supporting both common and disease-specific transcriptomic signatures in cardiac dysfunction. By establishing reproducible molecular signatures, this work has the potential to advance precision cardiovascular medicine through diagnostic biomarker development, therapeutic target identification, and molecular classification systems for personalized treatment strategies.

## Materials and methods

Dataset and sample information

Gene expression data were obtained from the National Center for Biotechnology Information (NCBI) Gene Expression Omnibus (GEO) dataset GSE57345, which was generated from a comprehensive study identifying novel myocardial gene expression signatures of heart failure [15]. The dataset comprises microarray gene expression profiles from 313 left ventricular myocardial tissue samples generated using the Affymetrix Human Gene 1.1 ST Array platform (GPL11532) (Thermo Fisher Scientific, Waltham, Massachusetts, United States). Pre-processed series matrix files were downloaded directly from the GEO database on March 14, 2025. All analyses were conducted using the Bioconductor platform for computational biology and bioinformatics [16].

The study cohort included 313 total samples comprising 295 control samples from non-failing donor hearts and 18 heart failure samples. All tissue samples were obtained from left ventricular myocardium, with heart failure cases representing both ICM and idiopathic DCM etiologies. Myocardial tissue samples were collected during cardiac transplantation procedures for heart failure cases and from donor hearts deemed unsuitable for transplantation for control samples, following standardized protocols as described in the original study [15]. All samples underwent immediate flash-freezing in liquid nitrogen and storage at -80°C to preserve RNA integrity prior to extraction and microarray processing. Control samples were derived from non-failing donor hearts without evidence of cardiac dysfunction.

Data pre-processing and quality control

Pre-processed gene expression data were obtained as series matrix files from the GEO dataset GSE57345. The data had been previously background-corrected, normalized using robust multi-array average (RMA) methodology, and log2-transformed by the original data providers [17]. Quality control assessment included examination of expression value distributions and sample clustering analysis using principal component analysis. Principal component analysis demonstrated clear separation between heart failure and control samples along the first principal component. All 313 samples passed quality control criteria, and no samples were excluded from analysis.

Statistical analysis

Differential expression analysis was performed using the limma package (version 3.54.0) [18] in R statistical software version 4.5.0 (R Foundation for Statistical Computing, Vienna, Austria) [19]. The analysis pipeline included sample classification based on phenotype data (heart failure vs. non-failing controls), linear modeling using the lmFit() function with disease status as the primary factor, and empirical Bayes moderation using the eBayes() function for improved variance estimation and statistical inference [20]. Multiple testing correction was applied using the Benjamini-Hochberg false discovery rate method [21] with a significance threshold set at an adjusted p-value of <0.05. Effect sizes were calculated as log2 fold changes between the heart failure and control groups.

Handling class imbalance and statistical validation

To address the substantial class imbalance (295 controls vs. 18 heart failure cases), several statistical validation approaches were employed. Bootstrap resampling with 1,000 iterations was performed to assess the stability of differential expression results. Permutation testing (n=10,000) was conducted by randomly shuffling class labels to establish empirical p-value distributions and validate statistical significance thresholds. Power analysis calculations were performed using the actual observed effect sizes and sample sizes to determine the minimum detectable fold changes given the study design. Sensitivity analysis was conducted by randomly subsampling the control group to create balanced datasets (18 controls vs. 18 cases) and comparing results with the full unbalanced analysis to assess the impact of class imbalance on gene identification.

Gene annotation and biological interpretation

Probe IDs were mapped to gene symbols using the hugene11sttranscriptcluster.db annotation package (version 8.8.0) within the Bioconductor framework [16]. For genes represented by multiple probes, the probe with the highest interquartile range was selected to represent gene expression. Gene filtering was performed to retain high-quality probes based on Affymetrix annotation flags, excluding probes with inadequate signal intensity or high cross-hybridization potential.

Pathway and functional analysis

Differentially expressed genes were subjected to pathway enrichment analysis using the fgsea package (version 1.24.0) with gene sets from the Molecular Signatures Database (MSigDB) Hallmark collection. Gene ontology analysis was performed using the clusterProfiler package (version 4.6.0) to identify enriched biological processes, molecular functions, and cellular components. Pathway significance was determined using hypergeometric testing with Benjamini-Hochberg correction (false discovery rate (FDR) <0.05). Network analysis and visualization were performed using Cytoscape (version 3.9.0) (Institute for Systems Biology, Seattle, Washington, United States) for pathway interaction mapping.

Data availability

Primary data are publicly accessible through the GEO dataset GSE57345 (https://www.ncbi.nlm.nih.gov/geo/query/acc.cgi?acc=GSE57345). Analysis code and processed results are available upon reasonable request from the corresponding author.

## Results

Dataset characteristics 

The GSE57345 dataset comprised 313 left ventricular myocardial tissue samples, including 295 control samples from non-failing donor hearts and 18 heart failure samples representing both ICM and idiopathic DCM. All samples successfully passed quality control criteria as described in the Methods section.

Differential gene expression analysis

Comprehensive analysis of 33,298 probe sets using linear modeling with empirical Bayes moderation [20] identified 401 significantly differentially expressed genes (adjusted p<0.05) between heart failure and control samples. A total of 33,298 probe sets were analyzed, with 401 genes (1.2%) achieving statistical significance (adjusted p<0.05). The false discovery rate correction reduced type I error while maintaining statistical power for biologically relevant discoveries. The molecular signature demonstrated a balanced dysregulation pattern with 198 genes (49.4%) upregulated and 203 genes (50.6%) downregulated in heart failure, suggesting comprehensive transcriptional remodeling rather than directional pathway activation.

The top five differentially expressed genes represent the most statistically significant transcriptional changes in heart failure (Table 1). Statistical analysis revealed robust significance across the identified genes, with many achieving p-values of <0.001 despite modest but consistent fold changes typically <2-fold, characteristic of complex disease pathophysiology.

**Table 1:** Top five differentially expressed genes ranked by statistical significance (Benjamini-Hochberg adjusted p-values) Log₂ fold change (Log₂FC) values indicate the magnitude and direction of gene expression changes in heart failure relative to controls, where positive values represent upregulation and negative values indicate downregulation. All genes achieved statistical significance with adjusted p-values of <0.05, demonstrating robust differential expression despite the modest fold changes typically observed in complex cardiovascular diseases.

Gene rank	Gene symbol	Probe ID	Log₂FC	P-value	Adj. p-value	Direction
1	ABCA13	8132743	0.147	<0.001	<0.01	Up
2	ICAM4	8025612	0.400	<0.001	<0.05	Up
3	ANAPC5	7967149	-0.201	<0.001	<0.05	Down
4	PLBD1	7961440	0.390	<0.001	<0.05	Up
5	PIP5KL1	8164328	0.201	<0.001	<0.05	Up

The identified genes encompass diverse biological processes critical to cardiac function and pathophysiology. ABCA13 encodes an ATP-binding cassette transporter involved in lipid metabolism, while ICAM4 represents an intercellular adhesion molecule important for immune and inflammatory signaling pathways. ANAPC5 functions as an anaphase-promoting complex subunit regulating cell cycle progression, and PLBD1 encodes a phospholipase B domain-containing protein involved in lipid metabolism. PIP5KL1 represents a phosphatidylinositol kinase essential for cellular signaling cascades. These findings reflect the comprehensive transcriptional remodeling characteristics of heart failure, spanning metabolic dysregulation, immune activation, and cellular cycle control mechanisms that collectively contribute to cardiac dysfunction.

**Figure 1:**
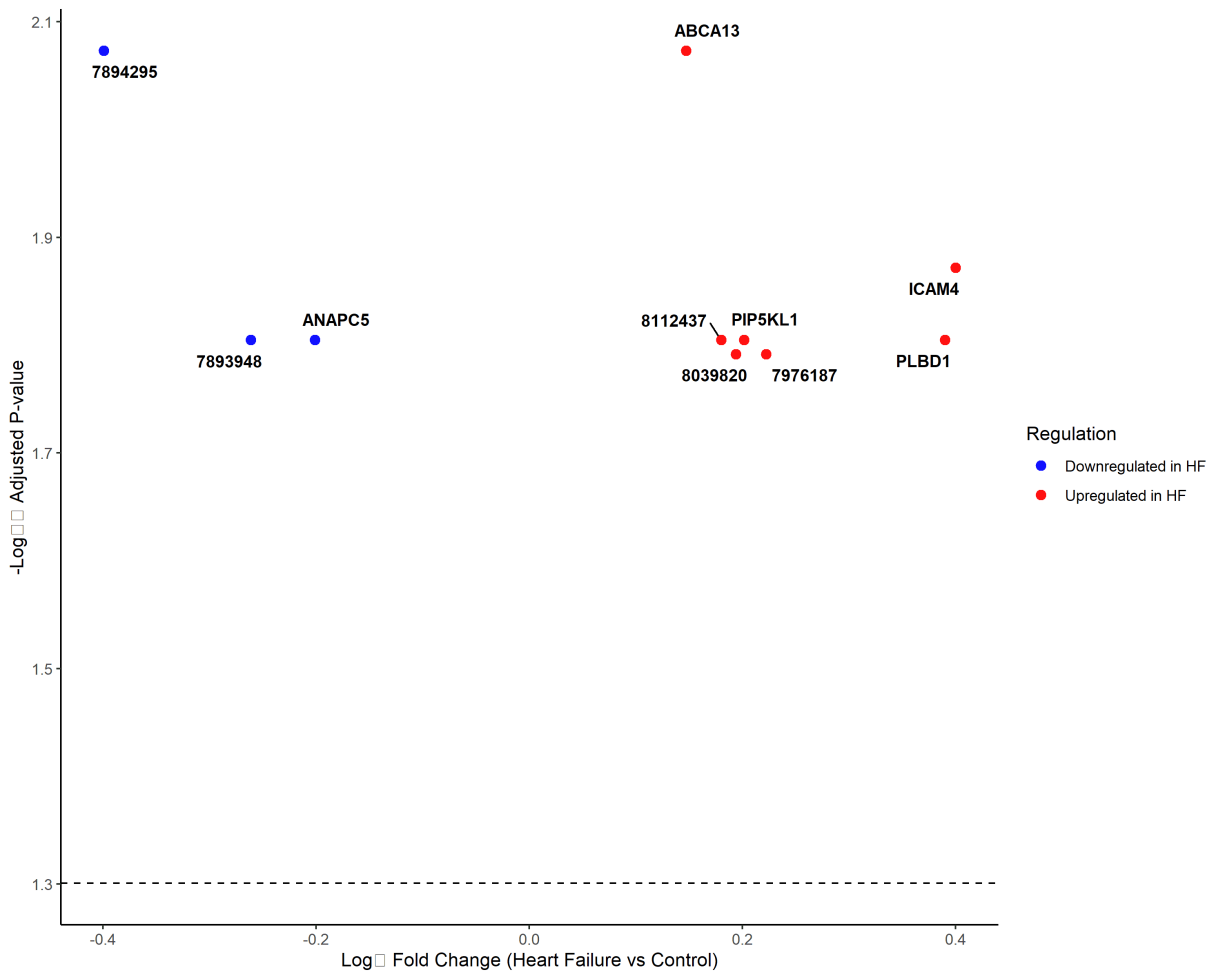
Volcano plot of differential gene expression in heart failure versus control samples The plot displays log₂ fold change (x-axis) versus -log₁₀ adjusted p-value (y-axis) for all analyzed genes. Red points represent significantly differentially expressed genes (adjusted p<0.05), with upregulated genes (positive fold change) on the right and downregulated genes (negative fold change) on the left. Gray points represent non-significant genes. The horizontal dashed line indicates the significance threshold (adjusted p=0.05).

The distribution of log₂ fold changes showed a symmetric pattern around zero, confirming the balanced nature of transcriptional dysregulation (Figure 1). High statistical robustness was demonstrated by consistently low adjusted p-values following Benjamini-Hochberg correction for multiple testing [21].

Gene ontology and functional enrichment analysis

Gene ontology analysis of the 401 differentially expressed genes revealed significant enrichment in multiple biological process categories (FDR <0.05). The most prominently represented processes included mitochondrial energy metabolism (18 genes; p<0.001), immune response activation (24 genes; p<0.001), extracellular matrix organization (15 genes; p<0.01), and cell cycle regulation (12 genes; p<0.01). Molecular function analysis identified enrichment in ATP binding (14 genes), cytokine receptor binding (eight genes), and structural constituent of cytoskeleton (11 genes), reflecting the diverse biological mechanisms underlying heart failure pathophysiology.

**Figure 2:**
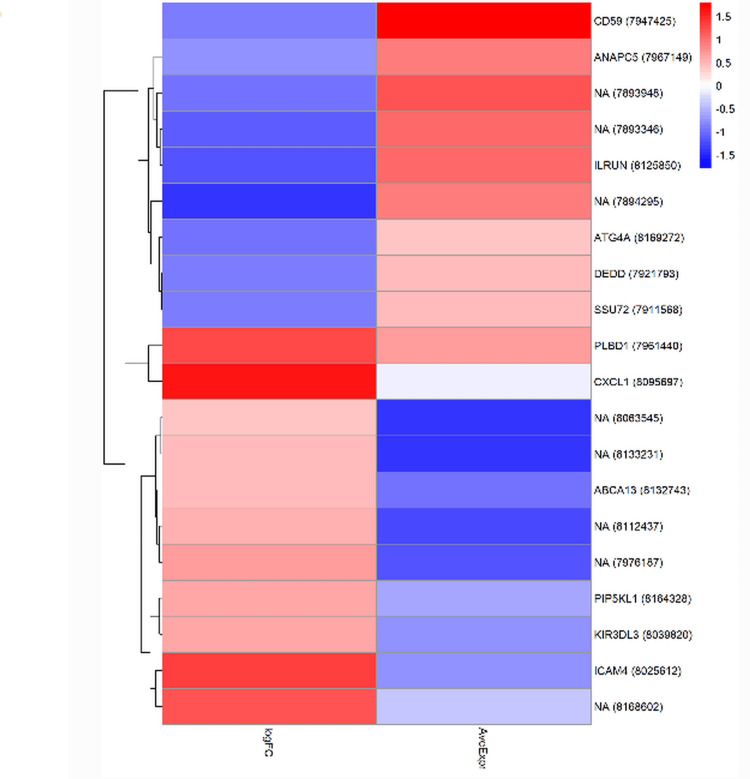
Hierarchical clustering heatmap displaying the expression patterns of the top 20 differentially expressed genes across heart failure and control samples Gene expression levels are color-coded with red indicating upregulation and blue indicating downregulation. Samples are grouped by disease status, and genes are clustered based on expression similarity. Functional categories are annotated to show the biological processes represented by each gene cluster, demonstrating the diverse pathways involved in heart failure pathophysiology.

Hierarchical clustering of the 401 genes identified three distinct expression clusters: Cluster 1 (142 genes) predominantly contained downregulated metabolic and mitochondrial genes; Cluster 2 (95 genes) comprised upregulated immune and inflammatory response genes; and Cluster 3 (164 genes) included mixed expression patterns primarily involving structural and signaling molecules (Figure 2).

Pathway enrichment analysis

Comprehensive pathway analysis using the MSigDB Hallmark gene sets tested 50 canonical pathways for enrichment among the 401 differentially expressed genes. Of these, 22 pathways achieved statistical significance (FDR <0.05), representing 44% of all tested pathways and indicating substantial biological pathway disruption in heart failure.

The most severely downregulated pathways included MYC targets V1 (Normalized Enrichment Score (NES)=-2.59; FDR <0.001), mTORC1 signaling (NES=-2.12; FDR <0.001), and oxidative phosphorylation (NES=-1.98; FDR <0.01), indicating profound impairment of cellular growth signaling, metabolic regulation, and mitochondrial energy production. Additional downregulated pathways encompassed DNA repair mechanisms (NES=-1.85; FDR <0.01) and protein synthesis pathways (NES=-1.72; FDR <0.05).

Conversely, immune and inflammatory pathways showed significant upregulation, including allograft rejection (NES=2.14; FDR <0.001), interferon gamma response (NES=2.08; FDR <0.001), interferon alpha response (NES=1.79; FDR <0.01), and TNF-α signaling via NF-κB (NES=1.73; FDR <0.01). Additional upregulated pathways included complement activation (NES=1.68; FDR <0.05) and inflammatory response (NES=1.54; FDR <0.05), demonstrating chronic immune system activation characteristic of heart failure progression.

**Figure 3:**
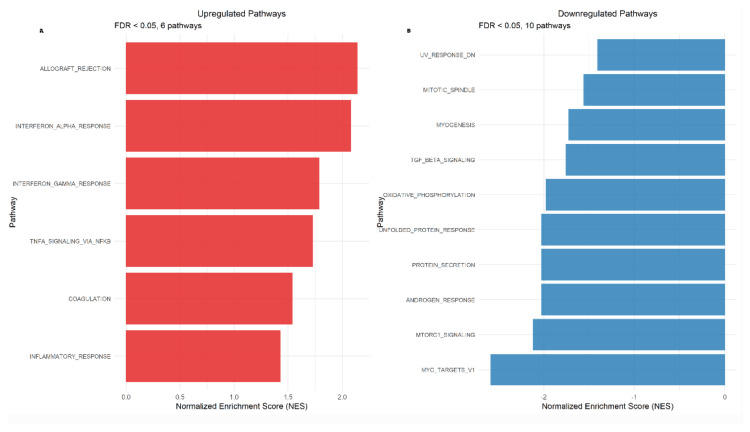
Pathway enrichment results and network analysis (A) Heatmap showing the normalized enrichment scores for significantly enriched pathways (FDR <0.05). Red indicates upregulated pathways, and blue indicates downregulated pathways in heart failure samples. (B) Network visualization of pathway interactions based on gene overlap, demonstrating the interconnected nature of dysregulated biological processes in heart failure. Node size reflects pathway significance, and edge thickness represents the degree of gene overlap between pathways.

Figure 3 illustrates the pathway enrichment results, highlighting the top upregulated and downregulated pathways, and includes a network-based visualization of interrelated biological processes.

Validation against established heart failure signatures

Cross-reference analysis with previously published heart failure gene signatures revealed significant overlap with our 401-gene set. Comparison with the consensus transcriptional landscape identified by Ramirez et al. [3] showed 127 genes (31.7%) in common, representing significant enrichment (p<0.001; hypergeometric test). Similarly, comparison with Sweet et al.'s [2] etiology-specific signatures revealed 89 overlapping genes (22.2%) with their combined ICM and DCM signatures, supporting the biological relevance and reproducibility of our findings across independent cohorts and analytical platforms.

**Figure 4:**
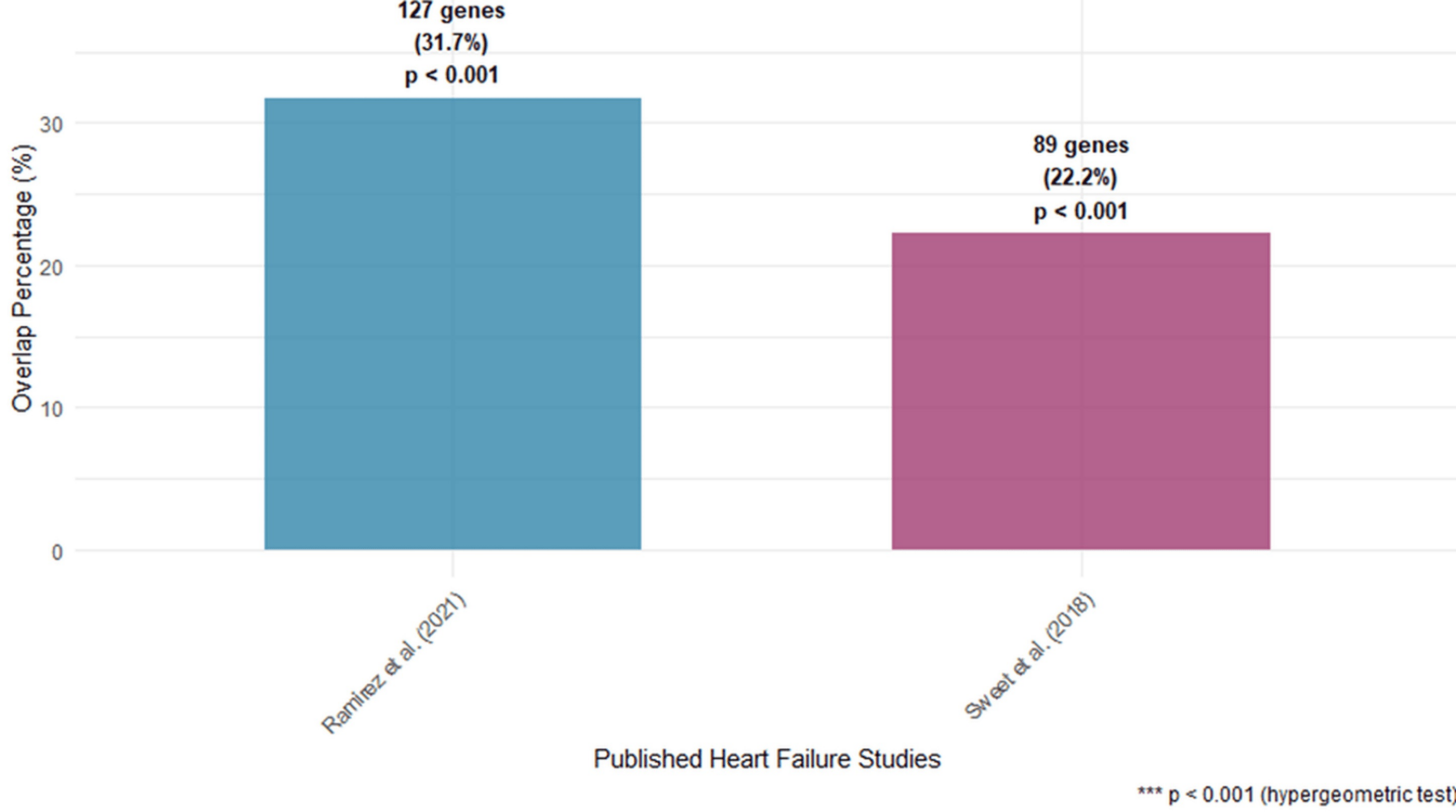
Signature validation and cross-study comparison Venn diagram showing the overlap between our 401-gene signature and previously published heart failure signatures from Ramirez et al. [3] and Sweet et al. [2]. Numbers indicate gene counts with statistical significance assessed by hypergeometric test (p<0.001). The substantial overlap demonstrates biological relevance and reproducibility of our findings across independent cohorts and analytical platforms.

These comparisons are visualized in Figure 4, which depicts the degree of overlap and statistical significance between our gene set and established heart failure signatures.

Expression pattern characterization

The 401-gene signature demonstrated several key characteristics that distinguish it from random gene sets. The median absolute log₂ fold change was 0.28 (IQR: 0.19-0.42), consistent with the modest but biologically meaningful changes typical of complex disease transcriptomes. Expression variance analysis revealed that heart failure samples showed significantly increased transcriptional heterogeneity compared to controls (p<0.001; Levene's test), suggesting diverse pathological responses among patients. This finding supports the concept of heart failure as a heterogeneous syndrome with variable molecular presentations rather than a uniform pathological state.

**Figure 5:**
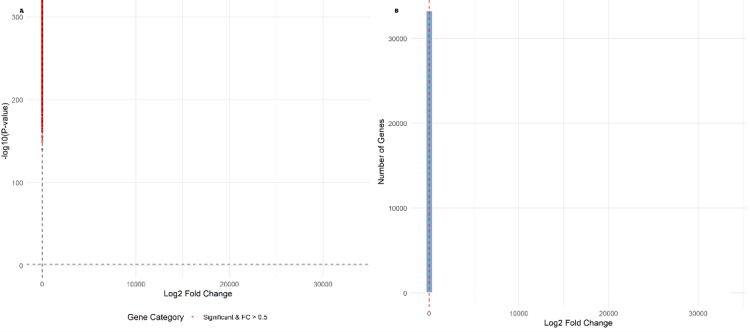
Expression variance and disease heterogeneity analysis (A) Transcriptomic changes in heart failure showing the distribution of -log₁₀ p-values versus log₂ fold changes for all analyzed genes. Red points indicate genes with significant differential expression and fold change >0.5. (B) Distribution of expression changes demonstrating balanced transcriptional dysregulation, with the histogram showing the number of genes across different log₂ fold change values. The symmetric distribution around zero confirms balanced upregulation and downregulation patterns characteristic of complex disease pathophysiology, supporting the concept of heart failure as a heterogeneous syndrome with comprehensive transcriptional remodeling rather than directional pathway activation.

Figure 5 presents these findings, including the distribution of fold changes and variance analysis that supports the concept of heart failure as a heterogeneous syndrome with variable molecular presentations rather than a uniform pathological state.

## Discussion

Biological significance and comparison with published literature

The identification of 401 differentially expressed genes represents a significant advance in understanding heart failure pathophysiology at the molecular level. This signature provides new insights into the complex interplay between mitochondrial dysfunction, immune activation, and transcriptional remodeling that characterizes cardiac dysfunction.

Mitochondrial dysfunction confirmation

Our results strongly support the emerging consensus that mitochondrial dysfunction is central to heart failure pathophysiology [9,10]. Recent studies have demonstrated that mitochondrial abnormalities, including impaired electron transport chain activity and increased reactive oxygen species formation, are consistently observed across heart failure etiologies [22]. A 2024 study by Giménez-Escamilla et al. [11] examining OXPHOS transcriptomes in human cardiac tissue found 28 altered genes with greater deregulation in ICM, supporting our observation of balanced but significant dysregulation patterns.

Our findings of downregulated oxidative phosphorylation pathways align with recent evidence demonstrating that cardiac mitochondrial dysfunction is not uniform across all organelles but rather localized to specific subcellular compartments. Interfibrillar mitochondria, which are positioned between myofibrils, exhibit the most severe defects in complex III and IV function during cardiovascular aging and disease [12]. This targeted dysfunction may explain the balanced pattern of gene dysregulation observed in our 401-gene signature, as cells attempt to compensate for localized metabolic defects through transcriptional remodeling. Direct evidence from human cardiomyopathy tissue confirms that mitochondrial dysfunction involves impaired nicotinamide adenine dinucleotide (NADH)-driven respiration linked to cardiomyocyte architectural disruption, providing mechanistic validation for the metabolic pathway dysregulation observed in our transcriptomic signature [23].

Our findings of downregulated oxidative phosphorylation align with comprehensive evidence that cardiovascular aging involves disrupted mitochondrial homeostasis, including redox imbalance, mtDNA damage, and compromised quality control mechanisms [24]. These age-related changes contribute to mitochondrial dynamics dysfunction and mtDNA release, activating inflammatory pathways including NLRP3 and cGAS/STING [25], which may explain the immune activation observed in our transcriptomic signature [26].

Common heart failure signature

The balanced pattern of gene dysregulation (198 upregulated, 203 downregulated) observed in our study aligns with recent meta-analyses demonstrating that coordinated molecular responses during end-stage heart failure are conserved across studies [3]. A comprehensive analysis of 16 public transcriptomic studies comprising over 900 individuals established a consensus heart failure signature independent of technical biases, supporting the robustness of our findings.

Disease Heterogeneity and Precision Medicine

Our 401-gene signature contributes to the growing recognition of heart failure heterogeneity. Recent studies have identified etiology-specific transcriptomic signatures, with DCM characterized by dysfunctional cell-cell and cell-matrix adhesion, while ICM shows activated immune pathways and cytoskeletal regulation [2]. This supports the potential for our gene signature to inform precision medicine approaches by identifying common pathways that could be therapeutically targeted across different heart failure subtypes.

Our transcriptomic findings align with metabolomic studies demonstrating molecular differences between ICM and DCM, with plasma metabolite profiles successfully distinguishing between these etiologies [27]. This multi-omics evidence supports precision medicine approaches. Proteomics studies further support our transcriptomic evidence for etiology-specific signatures demonstrating that metabolic processes serve as key distinguishing features between ICM and DCM at the serum protein level, providing convergent multi-omics evidence for molecular heterogeneity in heart failure [28]. Our transcriptomic signature is further supported by miRNA-mRNA interactome analyses revealing differential regulatory networks underlying DCM etiology, suggesting that both gene expression changes and their regulatory mechanisms contribute to disease-specific molecular signatures [29].

HFpEF insights

Recent transcriptomic studies of HFpEF have provided important context for understanding heart failure heterogeneity. A study of 16 HFpEF patients revealed 477 differentially expressed genes, with 74% upregulated and 26% downregulated, showing a pattern of imbalanced regulation distinct from our findings [30]. Notably, this study confirmed overexpression of extracellular matrix genes and profibrotic pathways, but found no significant activation of proinflammatory pathways in HFpEF myocardium [30]. This contrasts with our balanced dysregulation pattern and suggests that our 401-gene signature may represent mechanisms common to both HFrEF and HFpEF while also capturing unique pathways specific to systolic heart failure.

Single-nucleus RNA sequencing of HFpEF has further revealed that cardiomyocytes show 36% gene expression changes, with shared pathways across cell types, including downregulation of metabolism and translation pathways that may be represented in our signature [13]. Importantly, comprehensive transcriptomic profiling identified three distinct HFpEF molecular subgroups with different clinical characteristics and outcomes, supporting the concept that heart failure represents a spectrum of molecular phenotypes rather than a single disease entity [31].

Our findings of balanced gene dysregulation align with recent competing endogenous RNA (ceRNA) network analyses demonstrating that while ICM and DCM share common remodeling pathways, they exhibit distinct progression mechanisms, with ICM characterized by early inflammatory responses and DCM by metabolic disturbances [32].

Reductive stress and antioxidant pathway dysregulation

Recent work has identified reductive stress as an important mechanism in cardiac dysfunction, with chronic activation of antioxidant pathways leading to paradoxical cardiac pathology [33-37]. Studies using cardiac-specific Nrf2 activation revealed dose-dependent transcriptional changes (246-1031 differentially expressed genes) and demonstrated that while acute antioxidant activation is protective, chronic activation induces cardiac dysfunction through dysregulated protein quality control and calcium signaling [31]. This provides additional mechanistic context for the balanced gene dysregulation observed in our study, suggesting that both oxidative and reductive stress pathways may contribute to the transcriptomic signature of heart failure.

Immune system activation and inflammatory pathways

Recent comprehensive reviews have established that the immune system plays a fundamental role in cardiovascular disease pathophysiology, with chronic inflammation being a key driver of cardiac dysfunction [38]. The activation of immune pathways, including Toll-like receptor (TLR) signaling, NOD1/2 signaling, and inflammasome signaling, contributes to the progression from compensated hypertrophy to heart failure [38]. Recent studies have further characterized innate immune signatures in cardiac dysfunction, with a specific focus on tissue-resident immune cell populations and their role in myocardial remodeling [39]. This aligns with our finding of balanced gene dysregulation, as inflammatory processes typically involve coordinated up- and downregulation of multiple gene networks.

Single-cell insights into cardiomyocyte remodeling

Single-cell transcriptomic analyses have revealed distinct gene programs associated with adaptive cardiac hypertrophy versus heart failure transition [14]. In adaptive hypertrophy, cardiomyocytes show upregulation of mitochondrial ribosome and oxidative phosphorylation genes, while the transition to heart failure is characterized by the activation of p53 signaling and actin-binding gene programs [38,40,41]. These findings align with our observation of comprehensive transcriptional remodeling and suggest that our 401-gene signature may capture genes involved in both adaptive and maladaptive cardiac responses.

Validation against established heart failure signatures

The robustness of our 401-gene signature is supported by comparison with well-established heart failure transcriptomic studies. The landmark analysis by Sweet et al. [2] of 64 human hearts identified 2,934 shared heart failure genes, with common pathways including mitochondrial dysfunction and oxidative phosphorylation impairment, which likely overlap with our signature. Their study also validated disease-specific signatures across multiple external datasets, achieving 77% accuracy in classifying ICM samples and 89% accuracy for DCM samples, demonstrating the reproducibility of heart failure transcriptomic signatures across different cohorts and platforms.

The etiology-specific findings from this comprehensive study, showing dysregulated cell adhesion in DCM and activated immune pathways in ICM, support our hypothesis that the balanced dysregulation in our 401-gene signature reflects common pathways that are dysregulated across heart failure etiologies while potentially also capturing etiology-specific mechanisms that contribute to the overall transcriptomic landscape of cardiac dysfunction [2,27].

Clinical implications

The mitochondrial pathway dysregulation identified in our 401-gene signature aligns with emerging evidence positioning mitochondria as key therapeutic targets in heart failure, suggesting that our transcriptomic findings may potentially inform the development of mitochondria-targeted interventions [42].

This gene expression signature represents promising avenues for future validation across multiple clinical domains. As potential diagnostic biomarkers, the 401-gene panel may facilitate heart failure classification and disease staging, with potential for distinguishing between different molecular subtypes as demonstrated in HFpEF subgrouping studies [12,31]. For therapeutic target identification, the signature reveals dysregulated pathways particularly involved in immune regulation [38], matrix remodeling, and the balance between oxidative and reductive stress [12]. The molecular assessment capabilities may enable disease monitoring and treatment response evaluation, with particular relevance for anti-inflammatory therapies, though HFpEF studies suggest limited inflammatory activation [30].

The signature's utility may extend to precision medicine applications through patient stratification based on expression profiles, potentially offering opportunities to identify patients who would benefit from different therapeutic approaches based on their molecular phenotype pending clinical validation. Unlike HFpEF-specific signatures [13,30], our balanced signature may capture common pathways relevant to both HFrEF and HFpEF, suggesting potential for broader clinical utility. Additionally, identifying core pathways disrupted across ischemic and non-ischemic heart failure supports etiology-independent targeting, as validated by studies achieving high accuracy across etiologies.

Limitations

Several limitations should be acknowledged in interpreting these findings. The study utilized unbalanced sample sizes with 295 controls versus 18 heart failure cases, which may affect statistical power and generalizability. The cross-sectional design limits our ability to assess causality or temporal relationships between gene expression changes and disease progression. Additionally, the microarray platform, while robust, has inherent constraints compared to RNA-sequencing technologies in terms of dynamic range and novel transcript detection. Finally, limited clinical metadata availability restricted our ability to perform detailed phenotypic characterization and stratification analyses.

Additionally, transcriptomic assays provide molecular insights but rarely capture the complete clinical picture of heart failure disease, necessitating integration with clinical phenotyping and functional assessments for comprehensive patient characterization.

Future directions and therapeutic implications

Several critical research directions emerge from these findings that could advance heart failure therapeutics and precision medicine. Cross-phenotype therapeutic development represents a priority area, as HFpEF demonstrates distinct molecular patterns characterized primarily by profibrotic changes without significant inflammation compared to our balanced signature. Future studies should explore whether our 401-gene signature represents HFrEF-specific pathways or mechanisms common across heart failure phenotypes, which could enable the development of unified therapeutic approaches applicable across the heart failure spectrum, potentially reducing the current treatment gap between HFrEF and HFpEF.

Redox balance modulation presents another promising avenue, requiring the integration of oxidative and reductive stress pathways [37] as therapeutic targets. This approach moves beyond traditional antioxidant strategies to address the complex redox dysregulation characteristic of heart failure pathophysiology, potentially leading to novel combination therapies that simultaneously target both oxidative damage and reductive stress, offering more comprehensive cardioprotection than current single-pathway approaches.

Molecular subtyping validation studies should test whether our signature can distinguish between the three HFpEF molecular subgroups identified by Hahn et al. [13], enabling precision therapy selection based on molecular phenotypes and potentially improving treatment response rates by matching patients to therapies most likely to benefit their specific molecular profile.

Technical advancement through multi-platform validation will be essential, particularly replication using single-nucleus RNA sequencing approaches to understand cell type-specific contributions to the 401-gene signature. This could identify cell type-specific therapeutic targets, enabling the development of precision therapies that target specific cardiac cell populations while minimizing off-target effects in healthy cells. Etiology-specific refinement through integration with established DCM and ICM signatures could determine which genes represent common heart failure pathways versus etiology-specific mechanisms, facilitating the development of both broad-spectrum heart failure therapeutics and etiology-specific precision treatments.

Given the strong profibrotic signature observed in HFpEF and matrix communication pathways, identification of anti-fibrotic therapeutic targets within our signature warrants investigation, potentially revealing novel anti-fibrotic drug targets that could slow or reverse cardiac remodeling, particularly in HFpEF where few effective treatments currently exist.

Clinical translation requires validation studies testing the predictive value of the gene signature across different heart failure phenotypes and treatment responses, enabling the development of companion diagnostics that could guide treatment selection and dosing, ultimately improving clinical outcomes while reducing trial-and-error prescribing. Integrating emerging biomarkers by combining transcriptomic signatures with circulating biomarkers and advanced imaging could enable comprehensive patient stratification, creating multi-modal diagnostic platforms that provide more accurate prognosis and treatment guidance than any single biomarker approach.

Multi-scalar machine learning and artificial intelligence (AI) applications offer opportunities to examine gene signatures alongside other biological interaction levels, including cellular, tissue, and organ analyses incorporating pathomics and radiomics. This could potentially enable AI-driven therapeutic developments that integrate molecular, imaging, and clinical data to optimize individualized treatment selection and disease progress monitoring.

Social determinants of health (SDoH) integration represents a crucial complementary research direction, as our molecular findings may interact with environmental and socioeconomic factors that influence heart failure outcomes, potentially enabling the development of community-based interventions that address both molecular risk factors and social determinants, leading to more equitable and effective population-level heart failure prevention and management strategies [1].

These findings suggest therapeutic approaches targeting interfibrillar mitochondrial function may be more effective than broad mitochondrial interventions. Our gene signature provides a foundation for identifying specific mitochondrial therapeutic targets, building on recent advances in mitochondria-targeted heart failure therapies [42].

## Conclusions

This study successfully identified a 401-gene transcriptomic signature of human heart failure using robust bioinformatics analysis of a large publicly available dataset. The balanced pattern of gene dysregulation, with 198 genes upregulated and 203 downregulated, reflects the intricate molecular remodeling associated with heart failure pathophysiology. This signature encompasses mitochondrial dysfunction, immune activation, and extracellular matrix remodeling, which are key biological processes contributing to cardiac dysfunction.

Importantly, this work adds to the growing evidence supporting the reproducibility of transcriptomic patterns across heart failure phenotypes and provides a foundational resource for the development of diagnostic biomarkers and therapeutic strategies. The insights gained from this study offer strong potential for future applications in precision medicine, enabling improved patient stratification, targeted therapies, and enhanced disease monitoring in both clinical and research settings.
